# Totally Implantable Central Venous Access Port-Associated Bloodstream Infection Caused by Corynebacterium provencense: The First Case Report

**DOI:** 10.7759/cureus.71250

**Published:** 2024-10-11

**Authors:** Tetsuta Nishigaki, Katsushi Tanaka, Rika Kawasaki, Shunta Hashiguchi, Hideaki Kato

**Affiliations:** 1 Pharmaceutical Department, Yokohama City University Hospital, Yokohama, JPN; 2 Respiratory Medicine, Yokohama City University Hospital, Yokohama, JPN; 3 Infection Prevention and Control Department, Yokohama City University Hospital, Yokohama, JPN; 4 Clinical Laboratory Department, Yokohama City University Hospital, Yokohama, JPN; 5 Neurology and Stroke Medicine, Yokohama City University Hospital, Yokohama, JPN

**Keywords:** antimicrobial stewardship, bloodstream infection, central venous access port, corynebacterium provencense, vancomycin

## Abstract

*Corynebacterium* species are associated with healthcare-associated infections, specifically in device implantation. Here, we report a rare case of a 44-year-old man with a totally implantable central venous access port-related *Corynebacterium* infection. When he developed a fever on day four of admission, vancomycin treatment was initiated. On the 11th day, the totally implantable central venous access port was removed. *Corynebacterium provencense* was identified from two sets of blood cultures. Vancomycin treatment was continued for 14 days after port removal, and the patient was discharged home on the 47th day. Although *C.* *provencense* infection had not been previously reported in humans, it could be treated by port removal and vancomycin administration, as demonstrated in other reports on *Corynebacterium* infections.

## Introduction

The genus *Corynebacterium* consists of Gram-positive, catalase-positive, aerobic or aerobic-anaerobic, and generally nonmotile bacilli. *Corynebacterium provencens*e was first isolated from fresh stools of French patients with obesity in 2019 [1] and was named after Provence, the region in France where the strain was isolated [2]. *C. provencense* has only been reported as the causative pathogen of feline otitis [3], and its pathogenicity in humans is unknown. Here, we present the first reported case of totally implantable central venous access port (CV port)-associated bloodstream infection (BSI) caused by *C. provencense*.

## Case presentation

A 44-year-old man with primary central nervous system lymphoma (PCNSL) was rushed to Yokohama City University Hospital due to limb weakness and clonic seizure and was immediately admitted to neurology for treatment in 2023. He was diagnosed with PCNSL and received chemotherapy with rituximab and intrathecal methotrexate until 18 months ago. The patient’s medical history included diabetes mellitus, paroxysmal atrial fibrillation, hypertension, and glaucoma. His regular medications were levetiracetam (1,500 mg daily), bisoprolol tape (2 mg daily), famotidine (10 mg daily), sodium chloride (2 g daily), and brinzolamide/timolol combination ophthalmic suspension. He also received parenteral nutrition at home. His vital signs on admission were as follows: body temperature, 36.4°C; heart rate, 74 bpm in sinus rhythm; blood pressure, 115/77 mmHg; respiratory rate, 20 breaths/minute; and percutaneous oxygen saturation (SpO_2_), 96% with 1 L/min nasal cannula oxygen administration. The blood laboratory findings were as follows: white blood cell count, 10,000 cells/μL; C-reactive protein, 3.51 mg/dL (Table 1). Head computed tomography (CT) showed no significant changes compared to 6 months earlier, whereas chest CT was suspicious for mild bronchopneumonia. Almost a year ago, he underwent a tracheotomy to address the difficulty in expectoration of sputum due to worsening illness and reduce the risk of asphyxia. The patient was also treated for a CV port-associated BSI caused by methicillin-susceptible *Staphylococcus aureus* and underwent CV port removal and reimplantation nearly 6 months before admission.

**Table 1:** Laboratory findings on admission Alb: albumin, ALP: alkaline phosphatase, ALT: alanine aminotransferase, AST: aspartate aminotransferase, Baso: basophil, BUN: blood urea nitrogen, Ca: calcium, CK: creatine kinase, Cr: creatinine, CRP: C-reactive protein, Hb: hemoglobin, Hct: Hematocrit, K: potassium, LD: lactate dehydrogenase, Lymph: lymphocyte, Mono: monocyte, Na: sodium, Neut: neutrophil, Plt: platelet, RBC: red blood cell, T-bil: total-bilirubin, TP: total protein, WBC: white blood cell

Laboratory test	Value	Reference range
WBC	10,000	3,300-8,600 /μL
Neut	87.0	39.6-69.7 %
Lymph	3.0	19.5-47.0 %
Mono	10.0	2.2-10.4 %
Baso	0.0	0.0-1.8 %
RBC	3,530 × 10^3^	4,350-5,550 × 10^3^ /μL
Hb	11.2	13.7-16.8 g/dL
Hct	33.8	40.7-50.1 %
Plt	177 × 10^3^	158-348 × 10^3^/μL
TP	6.3	6.6-8.1 g/dL
Alb	2.8	4.1-5.1 g/dL
T-Bil	0.7	0.4-1.5 mg/dL
AST	45	13-30 U/L
ALT	43	10-42 U/L
LD	279	124-222 U/L
ALP	188	38-113 U/L
BUN	21	8-20 mg/dL
Cr	0.58	0.65-1.07 mg/dL
Na	134	138-145 mEq/L
K	4.9	3.6-4.8 mEq/L
Ca	8.4	8.8-10.1 mg/dL
CK	28	59-248 U/L
CRP	3.51	≤0.14 mg/dL

**Figure 1:**
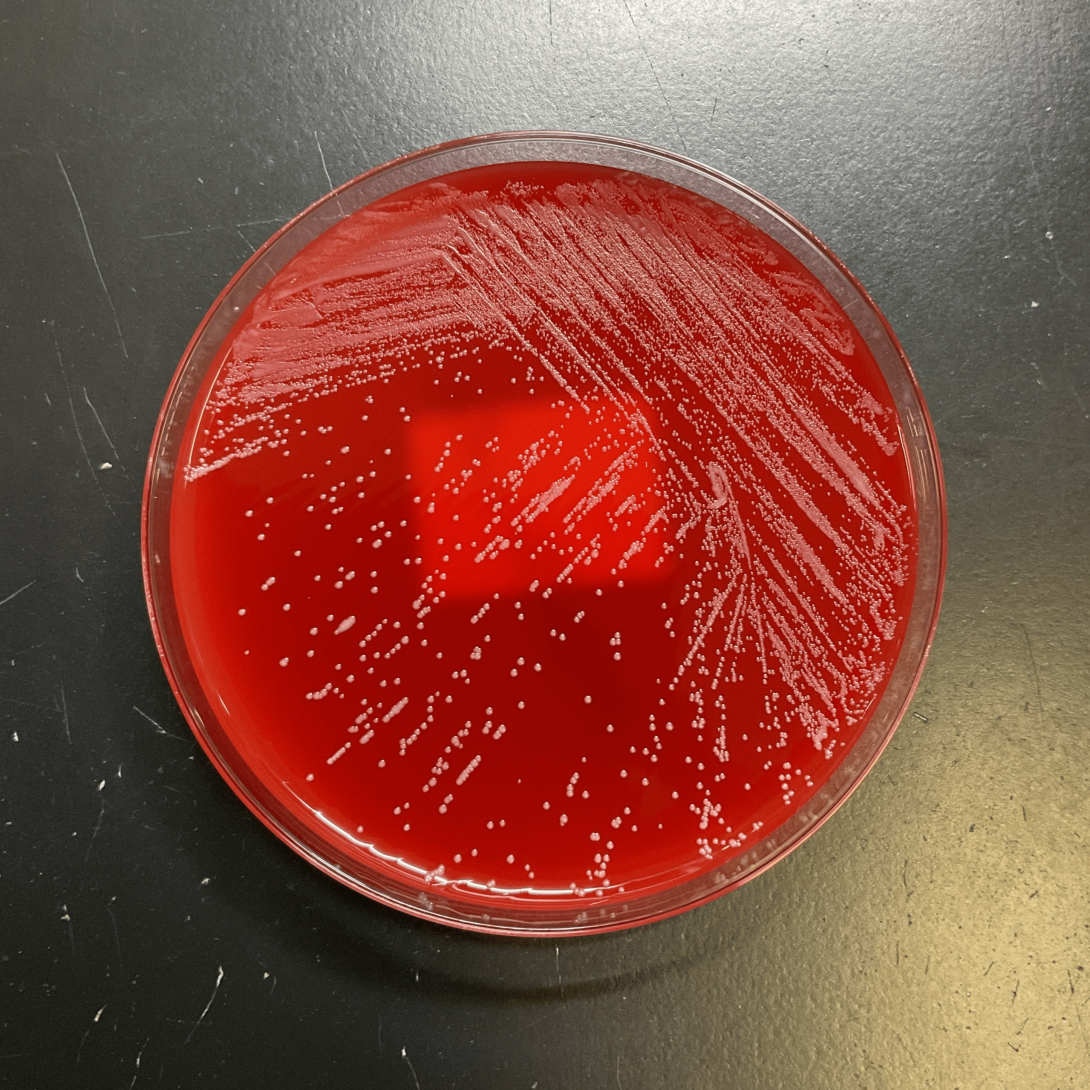
Isolation culture showing colony of Corynebacterium provencense on blood agar medium

On the first day of admission, fosphenytoin injection and levetiracetam injection were started, and the clonic seizure resolved on the same day. On day four after admission, the patient’s body temperature rose to 39.7°C. He underwent antibiotic treatment with vancomycin (VCM, intravenously; 1 g for the first dose and 0.6 g every 12 hours for the second and subsequent doses) for suspicion of CV port-associated BSI after two sets of blood samples were collected for culture. On day 5 after admission, following urine sample collection, the patient underwent antibiotic treatment with ceftriaxone (CTRX, intravenously; 2 g every 24 hours) for suspected concurrent urinary tract infection. On the evening of the same day, his heart rate increased to 160 beats per minute, his body temperature rose to 41.1°C, and he experienced chills. Furthermore, SpO_2_ dropped to 88%, and nasal cannula oxygen administration was started at 3 L/min. However, the patient’s breathing became irregular; thus, he was moved to the intensive care unit for ventilator management due to unstable respiratory status. After two sets of blood samples were collected again for culture, his antibiotic treatment was changed from CTRX to tazobactam/piperacillin (TAZ/PIPC, intravenously; 4.5 g every six hours). On day six after admission, gram-positive rods were detected from two sets of blood cultures submitted on day four. On day seven of admission, *Escherichia coli* (extended-spectrum β-lactamases-producing Enterobacterales) was identified from the urine culture. On day eight of admission, the patient’s SpO_2_ increased to 99% under room air, and his general condition improved, which warranted his transfer to the general ward. On day 11 of admission, the patient underwent CV port removal, and the catheter tip of the CV port was sent for culture analysis. No pus or other symptoms of infection were observed at the CV port placement site. Antimicrobial therapy with TAZ/PIPC was discontinued, while treatment for CV port-associated BSI was maintained with VCM alone. On day 13 after admission, *C. provencense *and *Corynebacterium* sp. (Table 2) were identified from the two sets of blood cultures submitted on day four (Figure 1).

**Table 2:** Minimal inhibitory concentrations for Corynebacterium provencense and Corynebacterium sp., interpretation according to The Clinical and Laboratory Standards Institute M45 guideline, third edition I: intermediate, MIC: minimal inhibitory concentration, R: resistant, S: susceptible

Corynebacterium provencense			*Corynebacterium* sp.		
Antimicrobial	MIC (μg/mL)	Interpretation	Antimicrobial	MIC (μg/mL)	Interpretation
Penicillin G	0.12	S	Penicillin G	>4	R
Ampicillin	0.25		Ampicillin	8	
Cefotiam	2		Cefotiam	>4	
Cefotaxime	2	I	Cefotaxime	>4	R
Ceftriaxone	1	S	Ceftriaxone	>4	R
Cefepime	1	S	Cefepime	>2	R
Cefozopran	0.5		Cefozopran	>4	
Cefditoren pivoxil	1		Cefditoren pivoxil	>1	
Meropenem	≤0.12	S	Meropenem	1	R
Clavulanic acid/Amoxicillin	≤0.25		Clavulanic acid/Amoxicillin	4	
Erythromycin	≤0.12	S	Erythromycin	0.25	S
Azithromycin	2		Azithromycin	0.5	
Clindamycin	1	I	Clindamycin	1	I
Minocycline	≤0.5		Minocycline	≤0.5	
Levofloxacin	≤0.25		Levofloxacin	>8	
Vancomycinancomycin	1	S	Vancomycinancomycin	0.5	S
Sulfamethoxazole-trimethoprim	≤0.5	S	Sulfamethoxazole-trimethoprim	1	S
Rifampicin	≤1	S	Rifampicin	≤1	S
Chloramphenicol	≤4		Chloramphenicol	≤4	

**Figure 2:**
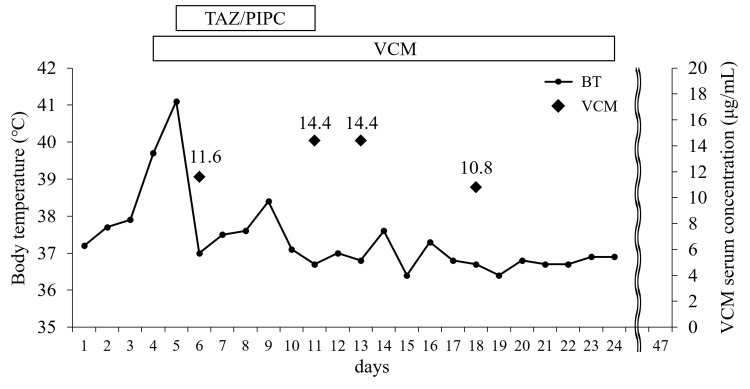
Treatment progress chart showing changes in body temperature and trough serum concentrations of intravenous vancomycin recorded over the treatment period Ceftriaxone was administered once on day five. TAZ/PIPC: tazobactam/piperacillin, VCM: vancomycin, BT: body temperature

*Staphylococcus caprae* was identified from one set of blood samples submitted for culture on day four, while *C. provencense* and *Corynebacterium* sp. were identified from one set of blood samples submitted on day five (the results of susceptibility testing were the same as on the fourth day). Gram-positive rods were detected from the tip of the CV port cultures submitted on day 11. However, the culture result was negative. Based on these results, a diagnosis of CV port-associated BSI caused by *C. provencense* and *Corynebacterium* sp. was made. The MALDI Bio-Typer (Nippon Becton Dickinson Co., Ltd., Tokyo, Japan) was used to identify the bacterial species, and the score for Rank 1 was *C. provencense*, with a score value of 2.11. Regarding *Corynebacterium* sp. identified from the same blood samples in which *C. provencense* was detected, *Corynebacterium* species with score values ​​of 1.70-1.99 were identified using the MALDI Bio-Typer. On day 19 of admission, the antimicrobial stewardship team (AST) proposed continued treatment with VCM for 14 days from the date of CV port removal. Two sets of blood samples were submitted for culture to determine the treatment efficacy on the same day. On day 24 after admission, two sets of blood samples submitted for culture on the 19th day tested negative, and the 14-day treatment with VCM was completed. The trough concentration remained between 10.8 and 14.4 μg/mL during the period of VCM administration (Figure 2). No recurrence of infection was observed during the remaining hospitalization period, and the patient was discharged home on day 47.

## Discussion

*C. diphtheriae* and *C. ulcerans* are the most common pathogenic *Corynebacterium* species associated with humans [4]. *Corynebacterium* species are found on the skin and in the respiratory tract as part of normal human flora [5]. They have low pathogenicity and are often contaminants when identified in clinical specimens [5]. In the present case, *C. provencense* and *Corynebacterium* sp. identified from two sets of blood cultures were considered the true causative organisms, not contaminants [6]. *Corynebacterium* species have been reported to form biofilms, which makes it easier for pathogens to adhere to catheters [7]. Yamamuro et al. [8] reported that the most common sources of infection with *Corynebacterium* species were catheter-related bloodstream infections (CRBSI). The patient had no suspected source of infection with *Corynebacterium* species except CV port-associated BSI based on the results of CT, cultures, and other examinations. Although our case did not meet the definitive diagnosis of CRBSI [6], for the reasons stated above, we considered *C. provencense* and *Corynebacterium* sp. to be the causative bacteria of CV port-associated BSI. The MALDI Biotyper identifies microorganisms using matrix-assisted laser desorption and the ionization-time of flight mass spectrometry to measure the proteins of an organism. The characteristic patterns of these proteins are used to identify a particular microorganism by matching the respective pattern with an open database to determine the identity of the microorganism down to the species level. A score value of 2.00 or higher indicates high-confidence identification, meaning high reliability at the bacterial species. A score value of 1.70-1.99 indicates low-confidence identification, meaning a match for the genus. Mass spectrometry was indispensable in the identification of the species in this case, and the frequency of clinical identification of *C. provencense* will increase in the future with the expected increased use of this analytical method. Infection is the most common complication after CV port implantation, with the incidence of CV port-associated infections ranging from 0.6% to 27%, or 2.81 cases per 1,000 days [9]. In addition, Liaw et al. [10] reported that the average time from CV port placement to the diagnosis of CV port-associated infection was 272 days (30-993 days), with wide interindividual variability. In our case, the infection occurred 180 days after implantation. Although human infections with some Corynebacterium species are known for transmission from animals [5], the patient had no history of animal contact. Although *C. provencense* has not been reported in humans except in feces, its identification as the causative agent of CV port-associated BSI in our case indicates that it may be a commensal skin bacterium like other *Corynebacterium* spp. The drug susceptibility of *Corynebacterium* varies greatly among species, and drug susceptibility must be confirmed prior to selecting antimicrobial agents [11,12]. *C. striatum*, *C. jeikeium*, and *C. resistens* may become highly drug-resistant; however, multidrug resistance among the infrequently recovered *Corynebacterium* species has been rarely observed [13]. Kato et al. [14] found that 97.6% of *Corynebacterium* strains were sensitive to VCM. Various reports have investigated the selection of therapeutic agents against *Corynebacterium* species [15]. Since *C. provencense* was found in this study to be sensitive to various antimicrobial agents other than VCM, targeted therapeutic agents other than VCM could also be considered. However, since infections in humans caused by *C. provencense* have not been reported, the AST at our institution proposed treatment with VCM as a reliable antimicrobial for targeting therapy. Although catheter-related *C. jeikeium* bacteremia may be successfully treated with vancomycin and without catheter removal [16], the principle of treatment for infection is the removal of the infection source; in our case, the CV port was removed on the 11th day of treatment. Ghide et al. [17] reported that the median duration of antimicrobial therapy for catheter-related BSI caused by *Corynebacterium* spp. is 12 days. In our case, the patient was treated for 14 days after catheter removal. Weakness and seizure are common symptoms of PCNSL [18]. In our case, we attributed these symptoms to PCNSL rather than to *C. provencense* bacteremia. Shim et al. [19] reported that the underlying hematologic malignancy was one of the independent risk factors of CV port-associated infection. We believed the possibility that underlying PCNSL might contribute to CV port-associated BSI caused by *C. provencense* in our case.

## Conclusions

We described a case of CV port-associated BSI caused by the rarely recovered *C. provencense*. Although infections caused by *C. provencense* in humans have not been previously reported, it was successfully treated by CV port removal and VCM administration, as recommended in previous reports on other *Corynebacterium* species. In our case, the trough concentration of VCM was 10-15 μg/mL, a relatively low concentration range that allowed treatment of *C. provencense*. Although it is to be noted that the patient had difficulty in communication, the predominant symptom of *C. provencense* bacteremia was fever, and no specific symptoms were observed. Further case reports are needed to determine whether immunocompromised status is important in the *C. provencense* infection. This report will hopefully contribute to knowledge on treatment for *C. provencense* infection.
